# Valerianate of Quinine

**Published:** 1846-04

**Authors:** 


*Valerianate of Quinine.*—In the *Memoriale della Medicina Contemporanea*, M. Galvani describes the following process for the preparation of the valerianate of quinine. Hydrated quinine, previously well washed to free it from all residue of the sulphate of ammonia, is to be triturated in a porcelain mortar, with a sufficient quantity of valerianic acid at 1000°, (the last product of the distillation of the valerianates of lime and soda, decomposed by sulphuric acid,) and constantly stirred until a perfect neutralisation of the salt is obtained, which may be ascertained by means of test papers. It sometimes happens that the valerianate produced by the supersaturation of the saline solution is precipitated, *en masse*, the solution itself remaining acid; at other times, if an acid be used of equal density, but obtained by another process, the operation does not succeed. In the first case a sufficient quantity of boiling distilled water must be added to dissolve the precipitated salt, a fresh quantity of the alkali being afterwards added to saturate the excess of the acid. The solution of the alkali in the acid being complete, it is to be placed in a water-bath, and, if requisite, so much organic acid added, not that the blue colour of reddened litmus paper be restored (which would risk the having a basic salt,) but in order that the blue may be slightly affected, without absolutely becoming red; by this means it is ascertained that the acid is only slightly in excess. The solution being effected, it is filtered, and set aside in a cold place; after the lapse of twenty-four hours a first product is obtained, of nipple-like form, of greater or less size, very white, and opaque. The mother-waters are then decanted, and the crystals set to drain. By exposing them in dry air, and neither in the sun nor on a stove, they will dry in a few hours, so that they can be detached entire with a spatula. Their desiccation may then be completed, by spreading them out on pasteboard in the open air. Although the crystals thus obtained have neither the hexahedral nor the octohedral form, and although the quinine in this process is not dissolved in alcohol, they are nevertheless composed of very pure valerianate of quinine, as M. Galvani has often ascertained by chemical analysis. Their odour and form, which differ from those of all the other salts of quinine, will be sufficient to distinguish them by.—*Lond. Med. Times.*

